# The Contrasting Relationships between Betaine and Homocysteine in Two Clinical Cohorts are Associated with Plasma Lipids and Drug Treatments

**DOI:** 10.1371/journal.pone.0032460

**Published:** 2012-03-02

**Authors:** Michael Lever, Peter M. George, Wendy Atkinson, Jane L. Elmslie, Sandy Slow, Sarah L. Molyneux, Richard W. Troughton, A. Mark Richards, Christopher M. Frampton, Stephen T. Chambers

**Affiliations:** 1 Canterbury Health Laboratories, Clinical Biochemistry Unit, Christchurch, New Zealand; 2 Pathology Department, Christchurch School of Medicine and Health Sciences, University of Otago, Christchurch, New Zealand; 3 Department of Medicine, Christchurch School of Medicine and Health Sciences, University of Otago, Christchurch, New Zealand; Brigham and Women's Hospital and Harvard Medical School, United States of America

## Abstract

**Background:**

Urinary betaine excretion positively correlated with plasma homocysteine in outpatients attending a lipid disorders clinic (lipid clinic study). We aimed to confirm this in subjects with established vascular disease.

**Methods:**

The correlation between betaine excretion and homocysteine was compared in samples collected from subjects 4 months after hospitalization for an acute coronary episode (ACS study, 415 urine samples) and from 158 sequential patients visiting a lipid disorders clinic.

**Principal findings:**

In contrast to the lipid clinic study, betaine excretion and plasma homocysteine did not correlate in the total ACS cohort. Differences between the patient groups included age, non-HDL cholesterol and medication. In ACS subjects with below median betaine excretion, excretion correlated (using log transformed data) negatively with plasma homocysteine (r = −0.17, p = 0.019, n = 199), with no correlation in the corresponding subset of the lipid clinic subjects. In ACS subjects with above median betaine excretion a positive trend (r = +0.10) between betaine excretion and homocysteine was not significant; the corresponding correlation in lipid clinic subjects was r = +0.42 (p = 0.0001). In ACS subjects, correlations were stronger when plasma non-HDL cholesterol and betaine excretion were above the median, r = +0.20 (p = 0.045); in subjects above median non-HDL cholesterol and below median betaine excretion, r = −0.26 (p = 0.012). ACS subjects taking diuretics or proton pump inhibitors had stronger correlations, negative with lower betaine excretion and positive with higher betaine excretion.

**Conclusions:**

Betaine excretion correlates with homocysteine in subjects with elevated blood lipids.

## Introduction

Betaine has central roles in mammalian metabolism both as an osmolyte and in the storage and transfer of one-carbon units [1], [2]. It is obtained from the diet, either directly or by the metabolism of dietary choline [1]. Disturbances in betaine metabolism have been linked to various diseases [1], [3], [4], but most often with vascular disease. Plasma betaine concentrations are low in patients with the metabolic syndrome [5] and in patients with lipid disorders [6], and evidence that betaine plays a role in the metabolic syndrome is growing [1]. An abnormal excretion of betaine, both high and low has been associated with diabetes and other diseases [7]. We previously reported that betaine excretion in subjects with lipid disorders correlated strongly with plasma homocysteine [6], especially in male subjects [8]. This implied that betaine loss was disturbing one-carbon metabolism in the study population, in which both plasma and urine betaine were major determinants of homocysteine. There is a plausible mechanism for such a connection, since betaine-homocysteine methyltransferase is a major determinant of homocysteine [9], [10], and therefore a betaine deficiency could be expected to cause elevated plasma homocysteine. However, we have not observed this relationship between betaine excretion and homocysteine in other populations including an Acute Coronary Syndrome cohort [11], and small studies of hip fracture patients [12] and stroke patients [13]. Elucidating the reasons for this difference could provide important information about the role of betaine in health and disease, and about the potential of dietary betaine intake for modifying disease risk.

A small sample of ambulant elderly subjects provided evidence that the positive correlation between urinary betaine and plasma homocysteine is characteristic of groups with elevated plasma lipids [14]. In the present study, we explored this relationship in a larger acute coronary syndrome cohort, and have compared these data with data from the lipid disorders clinic cohort. Our aim was to confirm the previous finding, and to identify factors that would define populations in which betaine excretion was related to plasma homocysteine.

## Methods

### Subjects

All study protocols were approved by the Canterbury Ethics Committee, and all subjects gave written informed consent.

The “ACS cohort” in this report was the previously described [11] sub-study using the Acute Coronary Syndrome (ACS) cohort. Inclusion criteria were as in De Lemos et al [15]. Exclusion criteria: Severe co-morbidity limiting life expectancy to less than 3 years. For the betaine sub-study fasting plasma samples were collected on 531 subjects at the four-month post-event follow-up visit to the clinic. Matching urine samples on 415 of these subjects were used in the present study.

The lipid clinic cohort has been previously described [6], [8]. Subjects (n = 158) attending the adult lipid disorders outpatient clinic at Christchurch Hospital, New Zealand were enrolled into the study. Subjects with diabetes were excluded.

In both studies fasting plasma and morning urine samples were collected on all subjects. Blood for homocysteine measurements was collected on ice. Samples were assayed for high volume laboratory tests within hours of collection, specimens for homocysteine, betaine and dimethylglycine assays were frozen at −16°C and assayed within two weeks.

Drug treatments and the diagnosis of diabetes were taken from clinical records.

### Laboratory methods

Betaine and *N,N*-dimethylglycine were measured in plasma and urine by high performance liquid chromatography (HPLC) by separation of their 2-naphthacyl derivatives on Merck Aluspher alumina columns [16], [17] with UV detection at 249 nm. Plasma homocysteine was measured by fluorescence polarization on an Abbott IMX Analyzer (Abbott Laboratories USA). Other biochemical measures in plasma and urine were all made by standard kit procedures in an International Accreditation New Zealand accredited laboratory, using an Abbott Aeroset Analyzer (Abbott Laboratories). Creatinine was measured using the Jaffé reaction, plasma cholesterol was measured by an enzymatic cholesterol oxidase reaction, triglycerides by enzymatic hydrolysis of triglycerides, both using Abbott reagents, and HDL cholesterol was measured by an enzymatic reaction using Roche reagents (La Roche Ltd, Switzerland).

Drug treatment and clinical diagnoses of diabetes were taken from the clinical records.

### Statistical analysis

Statistical analyses were carried out using SigmaPlot for Windows version 11.2 (Systat Software Inc), which incorporates SigmaStat.

In both sets of study data, urine betaine and *N,N*-dimethylglycine excretions were positively skewed, and in the ACS study plasma betaine, dimethylglycine and homocysteine concentrations were also positively skewed. Log-transformed data were normally distributed. Therefore all data were log transformed prior to conducting the correlation and regression analyses. Pearson's correlation coefficients were used to assess the strength of the associations between plasma and urine levels. Comparisons between cohorts and subgroups were undertaken using the non-parametric Mann-Whitney U tests. Multiple-linear regression models were used to determine the independent role of a number of putative predictors of plasma homocysteine. A two-tailed p-value<0.05 was taken to indicate statistical significance.

For estimating correlations, plasma betaine and *N,N*-dimethylglycine concentrations were adjusted for gender. In the ACS cohort, the mean male betaine concentration was 20.5% higher than the female mean; dimethylglycine was 11.7% higher in males. In the lipid clinic cohort, male plasma betaine concentrations were 34.4% higher than female; dimethylglycine was 18.6% higher in males. For adjustment, the raw concentrations for female subjects were multiplied by the appropriate factor to generate adjusted results with the same mean and standard deviations for both genders.

Correlation and regression statistics are reported with the number of valid data pairs quoted in the results, and with significance based on these. As a result of missing data these comparisons are usually based on fewer than the number of subjects in the ACS study. The significances of differences between correlation coefficients were evaluated using Fisher r-to-z transformations.

## Results

### Differences between cohorts

The lipid clinic cohort (for which diabetes was an exclusion criterion) was younger with a higher proportion of female subjects than the ACS cohort (**Table 1**). The most notable differences were the median plasma betaine concentrations and the plasma non-HDL cholesterol concentrations. The ACS cohort was also more heavily medicated (**Table 1**) and included subjects with diabetes. The upper quartile plasma betaine concentration of the lipid clinic cohort is less than the lower quartile plasma betaine in the ACS study, and the lower quartile of the non-HDL cholesterol concentration in the lipid clinic study is above the upper quartile of the ACS study.

**Table 1:** Comparison of study populations.

	ACS study		Lipid clinic study	
	Males	Females	Males	Females
Number	297	118	77	81
Age (median, total range)	66 (55–93)	72 (51–91)†	52 (43–59)***	56 (48–63)† ***
With diabetes (%)	16%	20%	0	0
BMI (median, IQ range)	26.3 (24.5–29.4)	27.4 (22.9–31.7)	28.4** (25.7–31.8)	27.2 (24.3–31.0)
Pl betaine µmol/L	42.1 (34.4–54.7)	38.0(30.5–44.1)†††	24.9 (19.2–31.9)***	20.0 (15.0–25.6)††† ***
Pl dimethylglycine µmol/L	3.7 (2.6–4.9)	3.2 (1.9–4.7)†	2.1 (1.5–2.7)***	1.8 (1.4–2.2)***
Pl total homocysteine µmol/L	12.5 (10.4–15.8)	12.3 (10.1–15.6)	9.8 (8.6–12.2)***	9.6 (7.7–12.2)***
Pl creatinine µmol/L	100 (89–110)	80 (71–100)†††	80 (70–90)***	70 (60–80)***
Pl urea mmol/L	6.5 (5.4–8.2)	6.4 (4.9–8.0)	–	–
Pl triglycerides mmol/L	1.50 (1.05–2.08)	1.47 (1.01–2.03)	2.25 (1.60–3.30)***	1.82 (1.27–2.35)†† **
Pl HDL cholesterol mmol/L	1.07 (0.89–1.25)	1.25 (1.06–1.50)†††	1.01 (0.87–1.30)	1.29 (1.10–1.55)†††
Pl non-HDL cholesterol mmol/L	3.0 (2.5–3.7)	3.3 (2.6–4.3)†	5.8 (5.1–6.6)***	5.5 (4.8–6.3)***
Betaine excretion	9.4 (6.0–18.1)	8.2 (4.4–17.7)	7.4 (5.0–13.9)*	8.1 (4.8–15.6)
Dimethylglycine excretion	2.9 (1.5–5.8)	2.4 (1.4–4.3)	3.8 (1.7–7.1)	3.2 (1.8–6.6)*
% on statins	89	81	17	21
% on ACE inhibitors	57	49	17	9
% on angiotensin II antagonists	8	10	0	0
% on diuretics	26	37	6	6
% on aspirin	94	96	12	9
% on clopidogrel	41	30	0	0
% on beta-blockers	88	81	9	9
% on calcium channel blockers	29	41	3	4
% on proton pump inhibitors	39	42	8	5
% current smoker	6	10	–	–

The ACS study data are on subjects who supplied urine samples. Median values with interquartile ranges unless otherwise stated. cr: creatinine. DMG: *N,N*-dimethylglycine. IQ: interquartile. Plasma (Pl) betaine, dimethylglycine and homocysteine in µmol/L; triglycerides and cholesterol fractions in mmol/L; excretions in mmol/mole creatinine. Significance (based on rank-sum test): difference between study populations,

* p<0.05;

** p<0.01;

*** p<0.001; difference between genders,

† p<0.05;

†† p<0.01;

††† p<0.001.

### Correlations between betaine, dimethylglycine and homocysteine

In the ACS population, plasma betaine, dimethylglycine and homocysteine concentrations approximated a log-normal distribution, so Pearson's correlation was estimated using log-transformed data. Plasma dimethylglycine concentrations correlated with both plasma homocysteine and plasma betaine concentrations (**Table 2**), but neither plasma betaine concentrations nor urine betaine excretion correlated with plasma homocysteine. The urinary excretion of dimethylglycine correlated with plasma dimethylglycine, but there was no correlation between urine and plasma betaine. When the genders were examined separately, the only significant difference was a stronger (p = 0.02) positive correlation between plasma dimethylglycine and homocysteine in females.

**Table 2:** Correlations between betaine, dimethylglycine and homocysteine in the two populations.

	Plasma dimethylglycine (µmol/L)	Plasma homocysteine (µmol/L)	Betaine excretion (mmol/mole)	Dimethylglycine excretion (mmol/mole)
A. ACS study				
Plasma betaine (µmol/L)				
r =	**+0.26**	−0.06	+0.027	−0.016
p =	**<0.00001**	0.15	0.59	0.75
n =	523	510	393	393
Plasma dimethylglycine (µmol/L)				
r =		**+0.25**	+0.05	**+0.16**
p =		**<0.00001**	0.33	**0.0013**
n =		510	393	393
Plasma homocysteine (µmol/L)				
r =			+0.019	+0.017
p =			0.70	0.74
n =			402	402
Betaine excretion (mmol/mole)				
r =				**+0.54**
p =				**<0.00001**
n =				415
B. Lipid clinic study				
Plasma betaine (µmol/L)				
r =	**+0.25**	−0.02	+0.08	−0.10
p =	**0.0017**	0.77	0.30	0.22
n =	158	158	158	158
Plasma dimethylglycine (µmol/L)				
r =		**0.0076**	+0.03	+0.03
p =		**+0.21**	0.69	0.67
n =		158	158	158
Plasma homocysteine (µmol/L)				
r =			**+0.35**	**+0.29**
p =			**<0.00001**	**0.0002**
n =			158	158
Betaine excretion (mmol/mole)				
r =				**+0.59**
p =				**<0.00001**
n =				158

Pearson's correlation coefficients calculated using log-transformed data. Plasma betaine and dimethylglycine concentrations corrected for gender difference. Excretions measured as mmole/mole creatinine. Significant correlations in bold.

For comparison, the data from the lipid clinic cohort was re-analyzed in exactly the same way, comparing log(homocysteine) with log(urinary excretions), despite a more normal distribution of betaine and homocysteine concentrations. The notable difference compared with the ACS cohort was that the urine betaine and dimethylglycine excretions correlated with plasma homocysteine. This correlation between log(urine betaine excretion) and log(plasma homocysteine) was stronger in the male subjects (r = +0.51, p<0.0001) than in the female subjects (r = +0.23, p = 0.039); difference in correlations, p = 0.048.

### Effect of diabetes

**Figure 1:**
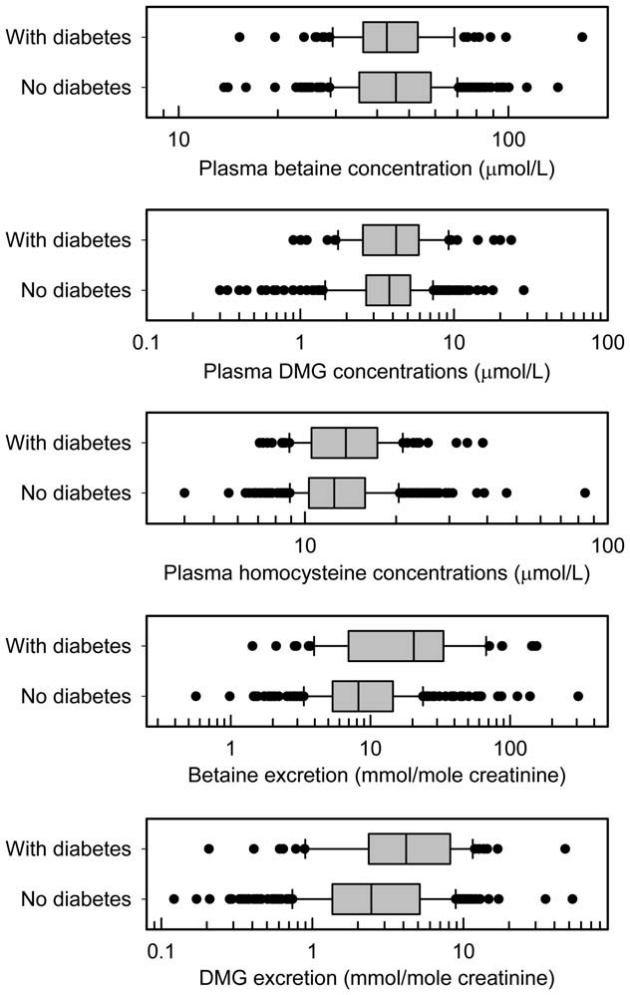
Differences in subjects with diabetes. ACS study subjects with (n = 90 plasma, 68 urine) and without (n = 425 plasma, 331 urine) diabetes; box shows median and interquartile ranges. Only the urinary excretions are significantly different (rank sum test), with excretions higher in diabetes, p<0.001 for betaine, p = 0.001 for dimethylglycine (DMG).

One difference between the cohorts was the inclusion of subjects with diabetes in the ACS cohort. Excluding subjects with diabetes did not change the differences between the cohorts shown in Table 1, and did not significantly change the correlations shown in **Table 2**. No significant correlations between betaine excretion and plasma homocysteine were detected in ACS subjects with (n = 67) or without (n = 321) diabetes, even though high excretions of both betaine and dimethylglycine were more common in subjects with diabetes (**Figure 1**).

### Different patterns associated with high and low betaine excretion

We have found that both high and low betaine excretion may be associated with disease [7]. Therefore, the above median betaine excretion and below median excretion subsets of the populations were analysed separately. In the low betaine excretion subsample of the ACS cohort (**Table 3**), there was a weak but significant negative correlation between betaine excretion and homocysteine; in the high betaine excretion subsample there was a non-significant trend for a positive association. Excluding subjects with diabetes had no effect on the below median excretion subgroup; the positive trend in the high excretion subgroup was strengthened by excluding subjects with diabetes (diabetes excluded, r = +0.16, p = 0.054). The corresponding results in the lipid clinic cohort showed a strong association between betaine excretion and plasma homocysteine in the above-median betaine excretion subjects (r = +0.42, n = 79, p = 0.0001) but no correlation was detected in the below median excretion subjects (r = −0.07, n = 79, p = 0.55).

**Table 3:** Correlations in ACS subjects split on median betaine excretion.

	Plasma dimethylglycine (µmol/L)	Plasma homocysteine (µmol/L)	Betaine excretion (mmol/mole)	Dimethylglycine excretion (mmol/mole)
A. Above median betaine excretion				
Plasma betaine (µmol/L)				
r =	**+0.23**	−0.07	−0.05	−0.015
p =	**0.0015**	0.36	0.50	0.84
n =	194	193	194	194
Plasma dimethylglycine (µmol/L)				
r =		**+0.23**	−0.03	**+0.27**
p =		**0.0016**	0.72	**0.0001**
n =		193	194	194
Plasma homocysteine (µmol/L)				
r =			+0.10	+0.07
p =			0.14	0.29
n =			194	194
Betaine excretion (mmol/mole)				
r =				**+0.39**
p =				**<0.00001**
n =				208
B. Below median betaine excretion				
Plasma betaine (µmol/L)				
r =	**+0.23**	−0.012	+0.015	−0.07
p =	**0.001**	0.87	0.84	0.33
n =	200	192	200	200
Plasma dimethylglycine (µmol/L)				
r =		**+0.24**	−0.12	+0.024
p =		**0.0009**	0.096	0.74
n =		192	200	200
Plasma homocysteine (µmol/L)				
r =			**−0.17**	−0.056
p =			**0.019**	0.44
n =			199	199
Betaine excretion (mmol/mole)				
r =				**+0.23**
p =				**0.0007**
n =				208

Pearson's correlation coefficients calculated using log-transformed data. Plasma betaine and dimethylglycine concentrations corrected for gender difference. Excretions measured as mmole/mole creatinine. Significant correlations in bold.

### Differences between subgroups based on plasma lipids, age and plasma betaine

Since the largest differences between the ACS cohort and the lipid clinic cohort were in non-HDL cholesterol, age, and plasma betaine the populations were divided on the basis of these factors into those subjects above the median and those below the median for each factor (**Table 4**). Plasma non-HDL cholesterol was most strongly associated with the correlation between urine betaine excretion and plasma homocysteine. In subjects with above median non-HDL cholesterol and above median betaine excretion there was a trend towards a positive correlation between betaine excretion and plasma homocysteine, whereas in the subjects with above median non-HDL cholesterol and below median betaine excretion there was a trend towards a negative correlation between betaine excretion and plasma homocysteine. These trends were not apparent in subjects with below median non-HDL cholesterol. The robustness of these trends was tested by estimating the correlations in the highest tertile and highest quartile of plasma non-HDL cholesterol. In subjects with above median betaine excretion, the Pearson's correlations (log-transformed homocysteine and urine excretion data) in the top tertile were r = +0.26 (p = 0.028) and in the top quartile r = +0.28 (p = 0.049). In subjects with below median betaine excretion, the correlations in the top tertile were r = −0.34 (p = 0.005) and in the top quartile r = −0.35 (p = 0.015). The correlations remained when subjects with diabetes were excluded; above median for both betaine excretion and non-HDL cholesterol, r = +0.25 (p = 0.032, n = 73) and below median for betaine excretion but above median for non-HDL cholesterol, r = −0.24 (p = 0.025, n = 87).

**Table 4:** Effects of plasma lipid, plasma betaine and age on correlations.

	Above median betaine excretion	Below median betaine excretion
*A. Correlations between plasma homocysteine and urine betaine excretion*		
Above median non-HDL-cholesterol	**+0.20 (0.045)**	**−0.26 (0.012)**
Below median non-HDL-cholesterol	+0.01 (0.96)	−0.09 (0.38)
Above median age	+0.11 (0.29)	**−0.23 (0.023)**
Below median age	+0.08 (0.44)	−0.04 (0.69)
Above median plasma betaine	+0.11 (0.28)	**−0.24 (0.018)**
Below median plasma betaine	+0.01 (0.90)	−0.05 (0.60)
*B. Correlations between plasma homocysteine and plasma betaine*		
Above median non-HDL-cholesterol	**−0.26 (0.011)**	−0.04 (0.72)
Below median non-HDL-cholesterol	+0.06 (0.57)	−0.01 (0.96)
Above median age	−0.19 (0.062)	−0.10 (0.34)
Below median age	−0.01 (0.93)	−0.04 (0.68)
Above median plasma betaine	+0.14 (0.18)	+0.08 (0.41)
Below median plasma betaine	**−0.26 (0.010)**	−0.02 (0.87)
*C. Correlations between plasma betaine and betaine excretion*		
Above median non-HDL-cholesterol	**−0.21 (0.044)**	+0.09 (0.37)
Below median non-HDL-cholesterol	−0.04 (0.72)	+0.03 (0.74)
Above median age	−0.08 (0.45)	+0.00 (1.00)
Below median age	+0.03 (0.76)	+0.01 (0.93)
Above median plasma betaine	+0.20 (0.053)	**+0.23 (0.023)**
Below median plasma betaine	−0.14 (0.16)	−0.09 (0.35)

Values are Pearson correlation coefficients between log-transformed variables, with significance (p) in parentheses after correlations. Significant correlations (p<0.05) in bold. Gender corrected plasma betaine. Urine betaine excretion measured as mmol betaine/mole creatinine. n = 101 (97 for plasma betaine).

Non-HDL cholesterol consistently did not correlate with homocysteine, neither in univariate correlations, nor when multiple regression models were estimated in these subgroups. In models with log(plasma homocysteine) as the dependent variable, and the independent variables plasma (gender adjusted) and urine betaine (both log transformed), non-HDL cholesterol and age, all the independent variables had variance inflation factors <1.2, with age appearing as the strongest predictor of homocysteine.

The lipid clinic study data was also divided into groups based on the medians of betaine excretion and plasma non-HDL cholesterol. In the above median betaine excretion and above median non-HDL cholesterol group (n = 39), the Pearson's correlation coefficient between plasma homocysteine and urine betaine excretion (both log-transformed) was r = +0.52 (p = 0.00066) whereas in the below median betaine excretion and above median non-HDL cholesterol group (n = 40) the corresponding correlation was not significant, r = +0.13 (p = 0.42).

### Drug therapy, betaine and homocysteine

In the ACS cohort, plasma betaine concentrations and betaine excretions were significantly different in subjects taking some medications (**Table 5**). As well as the previously reported [11] differences between subjects taking lipid lowering drugs, plasma betaine was also higher in subjects taking beta-blockers and lower in subjects taking angiotensin II antagonists. Apart from fibrates, no other drug treatment was associated with a significant difference in betaine excretion.

**Table 5:** Effect of drugs on plasma betaine and betaine excretion.

	Not taking drug				Taking drug			
Drug category	n	pl betaine	n	betaine excretion	n	pl betaine	n	betaine excretion
Statins	68	40.2	52	7.6	436	45.9**	349	9.0
Fibrates	493	45.2	392	8.8	12	33.0**	10	99.8***
Diuretics	359	45.2	282	9.2	144	44.1	116	8.1
Beta blockers	78	39.8	56	8.6	426	46.1**	343	9.0
ACE inhibitors	236	45.0	181	9.1	268	45.1	218	8.8
Angiotensin II antagonists	460	45.3	362	8.8	40	42.0*	34	10.7
Clopidogrel	308	45.3	248	8.7	196	44.7	151	9.1
Calcium channel blockers	344	45.3	270	8.5	160	43.3	129	9.8
Proton pump inhibitors	298	43.7	242	8.5	207	46.5	158	9.7
Current smokers	474	45.3	372	8.8	31	37.6	28	12.1

* p<0.05;

** p<0.01;

*** p<0.001 compared with subjects not taking the medication.

Median plasma betaine (corrected for gender): µmol/L; betaine excretion: mmol urine betaine/mole creatinine.

Because medication was one of the main differences between the ACS cohort and the lipid clinic cohort (**Table 1**), correlations were estimated between plasma homocysteine, plasma betaine and betaine excretion, and plasma dimethylglycine and dimethylglycine excretion, in the above and below median betaine excretion groups of the high plasma lipid subgroups of the ACS cohort that were being treated with different medications. These were compared with subgroups not being treated (**Table 6**). There were too few subjects being treated in the lipid clinic cohort for a comparable analysis. To enhance comparability with the lipid disorders clinic cohort, the correlations are reported with subjects with diabetes excluded, but in all cases there were only minor and insignificant differences when these subjects were included. The trends for a positive correlation between high betaine excretion and homocysteine, and for a negative correlation between low betaine excretion and homocysteine, were robustly reproduced in different subgroups. The positive correlation between plasma homocysteine and betaine excretion, in subjects with above median betaine excretion, increased in subjects not being treated with ACE inhibitors or clopidogrel, and in those not receiving proton pump inhibitors. The negative correlation between low betaine excretion and homocysteine was greater insubjects treated with diuretics (p = 0.020) or proton pump inhibitors (p = 0.044). There were too few subjects not taking aspirin for a similar comparison (**Table 1**), though omitting these subjects had a negligible effect on the results. A highly significant effect was seen with subjects treated with angiotensin II receptor antagonists; there were too few of these to analyse the data in the same way, but in the group of all subjects without diabetes being treated with these drugs (n = 24) the correlation between log(urine betaine excretion) and log(plasma homocysteine), Pearson's r = +0.77 (p = 0.00001); if subjects with diabetes are included (n = 34), r = +0.44 (p = 0.009).

**Table 6:** Dependancy of correlations on medication.

Correlations between plasma homocysteine and betaine excretion in:	Above median betaine excretion			Below median betaine excretion		
	n	r	p	n	r	p
*Subset based on drug treatment:*						
All subjects without diabetes	73	**+0.25**	**0.032**	87	**−0.24**	**0.025**
No diuretics	52	+0.21	0.13	61	+0.12	0.36
With diuretics	17	+0.11	0.67	26	**−0.42**	**0.033**
No beta blocker	7	+0.57	0.18	14	+0.12	0.67
With beta blocker	65	**+0.26**	**0.035**	73	**−0.30**	**0.010**
No ACE inhibitor	38	+0.13	0.42	36	**−0.36**	**0.036**
With ACE inhibitor	34	**+0.43**	**0.011**	51	−0.15	0.30
No clopidogrel	43	+0.11	0.49	58	−0.15	0.26
With clopidogrel	29	**+0.46**	**0.012**	29	**−0.37**	**0.050**
No calcium channel blocker	46	+0.10	0.52	61	−0.19	0.14
With calcium channel blocker	26	+0.39	0.051	26	−0.21	0.31
No proton pump inhibitor	45	**+0.32**	**0.032**	52	+0.01	0.95
With proton pump inhibitor	28	+0.17	0.38	35	**−0.42**	**0.011**

All data on subjects with above median plasma non-HDL-cholesterol and with subjects with diabetes excluded. Pearson's correlation coefficients between log (plasma tHcy) and log(urine betaine/creatinine). Bold significant (p<0.05).

To a lesser extent, relationships other than that between homocysteine and betaine excretion were changed in subgroups treated with some drugs (Table 6). In six sub-groups there was a significant (p<0.05) negative correlation between plasma betaine and homocysteine. The strongest correlation was in subjects treated with calcium channel blockers and above the median for both betaine excretion and for plasma non-HDL cholesterol, r = −0.51 (n = 34, p = 0.002).

## Discussion

In our previous study of subjects attending a lipid disorder clinic, we found a strong association between plasma homocysteine and betaine excretion [6], and the urinary betaine excretion was the strongest determinant of plasma homocysteine in the male subjects [8]. We were therefore surprised when similar associations were not seen in several subsequent studies, some briefly described elsewhere [13], [14] and some unpublished [12], [13]. We have shown here that our previous results [6], [8] were serendipitous consequences of the population we sampled. One cohort that did not show the expected correlation was the ACS study group, who were stabilized subjects recruited at a clinic visit approximately four months after hospital admission following a cardiovascular event. These differed from the lipid disorders clinic subjects in that almost all were receiving standard cardiovascular medications including lipid lowering agents. Few of them had elevated plasma lipids when studied, whereas in contrast, the subjects recruited from the lipid clinic required specialist outpatient management for disordered lipid profiles. The differences were large; the bottom quartile of non-HDL cholesterol in the lipid clinic subjects was above the top quartile in the ACS cohort. We found a positive correlation between an elevated betaine excretion and plasma homocysteine in sub-groups of the ACS cohort with elevated plasma lipids, with non-HDL cholesterol a strong marker of this (with the correlation increasing with increasing non-HDL cholesterol). In addition, in the total ACS population, there was a confounding trend for low betaine excretion to negatively correlate with plasma homocysteine; this trend was weak or absent in the lipid disorders cohort, and was stronger in the subsets of ACS subjects treated with drugs such as diuretics and proton pump inhibitors that were more commonly prescribed to ACS subjects. The median plasma betaine concentrations in both sexes of the ACS population were close to normal [1] and were much higher than those in the lipid disorders cohort. Plasma betaine is not independent of plasma lipids and an inverse relationship that has been previously described [5] was present in the ACS population in this study [11]. The other major difference between the two study populations is age, which is an independent determinant of plasma homocysteine and does not explain the difference; in contrast, although the non-HDL cholesterol concentration efficiently defines sub-groups in which betaine excretion determines plasma homocysteine, the lipid concentration itself did not correlate with plasma homocysteine.

When we examined the cohort of subjects without diabetes who were attending a lipid disorders clinic, we expected that this group would have a high proportion of subjects with pre-diabetic conditions, and we found in this group that betaine (both plasma concentrations and urinary excretion) was a major determinant of plasma homocysteine concentrations [6], and in male subjects the urinary betaine excretion was the strongest determinant of homocysteine, stronger than folate [8]. In this mainly folate-replete population we concluded that homocysteine was a marker of betaine deficiency, and found that betaine loss appears to be associated with vascular risk [6]. Homocysteine is a well-known risk factor for cardiovascular disease [18]–[20]. However, studies in which folate supplementation decreased the mean plasma total homocysteine concentrations have not shown a corresponding decrease in subsequent vascular events [21]–[23]. There has been considerable recent discussion of this paradox and a number of suggested resolutions put forward, which is not surprising given the strong association between homocysteine and both cardiovascular disease and heart failure [24], [25]. However, it is usually overlooked that betaine may be the major determinant of plasma homocysteine [1], [26] which is largely controlled by the liver enzyme betaine-homocysteine methyltransferase [9], [10], and most discussion is focussed on the role of folate and vitamin B_12_ mediated homocysteine lowering. There is now a growing awareness of importance of betaine in human health [1]–[4]. It has dual roles as a key osmolyte and as a source of methyl groups, and tissue concentrations are typically an order of magnitude higher than circulating blood concentrations [27]. More than 20% of patients with diabetes mellitus excrete abnormal amounts of betaine [28], [29]. Since betaine is normally metabolized, and this metabolism inevitably involves the conversion of homocysteine to methionine [1], it seemed plausible that betaine loss in the urine could be both a major determinant of plasma homocysteine concentrations and be itself pathogenic, and our initial observation supported this model.

Here we ask why this association between betaine excretion and homocysteine was not subsequently observed in the ACS study. This population, and the lipid disorders clinic cohort, were quite different and neither is typical of the general population. The contrast in control of homocysteine between the cohorts is most strongly associated with the large difference in the plasma lipid profiles of the two populations, and another factor is the difference in the degree of medication of the subjects. The correlation between urine betaine excretion and plasma homocysteine increases in subsets of the present study with higher lipids, but the number of subjects becomes too small to detect significant correlations at lipid concentrations well below those in the earlier study. A confounding factor is the presence of subjects with abnormally [1] low betaine excretions, as seen in previous studies [7]: these also appear to be high risk subjects (unpublished data). Plasma non-HDL cholesterol appears to be a marker of subjects in whom betaine excretion is related to plasma homocysteine, but itself is not statistically a determinant of homocysteine. These results confirm observations made in a smaller study of ambulant elderly subjects [14]. It is possible that drugs affect betaine and homocysteine metabolism, but the differences we observed may reflect differences between the sub-populations that are prescribed these drugs; the cross-sectional design means that we cannot distinguish these alternatives, but both are plausible. In the case of fibrates it is more likely to be an effect of the drug [11], [13], and the same might be true of the angiotensin II antagonists reported here, but in many cases it is likely that the results reflect the selection of patients who are treated with that drug. The correlations seen with low betaine excretions is obviously associated with subjects being treated with diuretics or proton pump inhibitors, but it does not necessarily follow that the drug treatment is responsible for the effect, and since most subjects are on multiple drugs, the effects suggested by Table 6 are not independent. In the metabolic syndrome where high blood pressure is common, subjects are likely to be treated with one or more of the drugs listed in tables 5 and 6. Subjects with the metabolic syndrome are also likely to have elevated lipids, and we have previously suggested that these are subjects who are likely to become betaine deficient [1]. Age itself may not be a critical factor, but further investigation would be justified into the diseases associated with age, and the effects of medication used to treat these. We acknowledge that in our analyses we have made ad-hoc multiple comparisons within subgroups, and therefore type I errors are not excluded. However, the consistency of the patterns of correlations suggests robustness in the results, but a cautious interpretation is required.

In conclusion, the results of the two studies can be reconciled, but the apparent contradictions are a warning about generalizations based on a selected patient population. The reconciliation confirms the association between betaine and lipid metabolism, and is consistent with a role for betaine in the metabolic syndrome. These cross-sectional studies suggest a number of hypotheses to be tested. The results are consistent with our previous suggestions [1] that betaine plays an important role in the metabolic syndrome, and this could be tested by longitudinal studies of supplementation. The cross-sectional results suggest that betaine deficiency is most apparent in subjects with elevated plasma lipids, and the hypothesis that there is a causal connection needs to be tested; it cannot be excluded that it is the elevated lipid that affects betaine, since in rats a high lipid diet appears to cause an elevation in betaine excretion [30]. As for mechanisms, the osmolyte role of betaine needs to be considered as well as its role as a methyl donor, and this is also consistent with the possibility that betaine metabolism is affected by drugs that modify the renin-angiotensin system. Studies to identify such connections may suggest improved therapeutic strategies that could benefit those subjects with the metabolic syndrome who have elevated lipids and increased vascular risk.
